# Language Preferences in the Dutch Autism Community: A Social Psychological Approach

**DOI:** 10.1007/s10803-023-05903-0

**Published:** 2023-02-09

**Authors:** Renate Bosman, Jochem Thijs

**Affiliations:** Utrecht University, Padualaan 14 2, 3584 CH Utrecht, The Netherlands

**Keywords:** Autism, Identity-first language, Person-first language, Identification, Age of diagnosis

## Abstract

This research examined the preference for identity-first language (IFL) versus person-first language (PFL) among 215 respondents (*M*_*age*_ = 30.24 years, *SD* = 9.92) from the Dutch autism community. We found that a stronger identification with the autism community and a later age of diagnosis predicted a stronger IFL preference and a weaker PFL preference. Both effects were mediated by the perceived consequences (justice to identity, prejudice reduction) of PFL. Participants’ own explanations were in line with these statistical analyses but also provided nuance to the IFL-PFL debate. Our results are consistent with the Social Identity Approach (Reicher et al., 2010) and Identity Uncertainty Theory (Hogg, 2007) and demonstrate the value of a social psychological approach to study disability language preferences.

**Supplementary Information:**

The online version contains supplementary material available at 10.1007/s10803-023-05903-0.

## Introduction

*We’ll often say that if you have to put the word ‘person’ first to remind yourself that we’re people, you really have a problem, not us (…). Because if you have to go through linguistic gymnastics to remind yourself that we’re people, you already didn’t believe we were people.*

The above extract comes from a news article on communication about disabilities (Malone, 2021, p.1) and quotes autism advocate Lydia X. Z. Brown. Their remark refers to an ongoing discussion on the use of person-first language versus identity-first language when describing and addressing people with disabilities, such as autism. Person-first language (PFL) is based on the reasoning that people are more than their disabilities, and puts the person before the disability (e.g., “person with autism”). It is recommended by many governmental organizations and institutions including the Centre for Disease Control and Prevention (CDC), the UK government, the APA, and the university of the authors (ODR, n.d.; UK government, 2022; CDC, 2022), yet those with disabilities themselves do not always agree with this recommendation. When it comes to autism, the use of PFL is supported by some in the community but criticized by others (such as Lydia X. Z. Brown) who prefer identity-first language (IFL) instead. In contrast to PFL, IFL emphasizes that one’s disability is often a central part of one’s identity which deserves to be mentioned before one’s personhood (e.g., “autistic person”).

The debate on how to appropriately refer to people from the autism community is a difficult and sensitive one (see Botha et al., 2021; Lei et al., 2021; Vivanti, 2020; Dwyer, 2022), which needs to be carefully conducted and cannot be settled by empirical research. However, research can make a meaningful contribution by systematically examining the perspectives of those directly affected by this language, and by investigating the differences in language preferences *within* the autism community. The present study tried to complement the small body of quantitative literature on this topic, by questioning a sample of 215 adults diagnosed with autism and using a social psychological approach to predict their evaluations of PFL and IFL. Whereas most of the social science research on autism is conducted in Anglophone countries (Cascio, 2015), our study was conducted in the Netherlands where the research on inclusive language about disabilities has started only a few years ago (Buijsman et al., 2022).

### Models for Characterizing People with Disabilities

Two major conceptual models for thinking about disabilities are the *social model* and the *minority model*. Both of them have implications for language use, and they can be contrasted to earlier models, such as the medical model, which (primarily) sees disabilities as problems (Dunn & Andrews, 2015). The social model considers disabilities as neutral characteristics and focuses on the social barriers faced by those who have them, including prejudice and discrimination. The model proposes that individuals should not be defined in terms of their disabilities, because this could objectify them and promote stereotypes and essentialism (Bloom, 2010), but also because everyone experiences their disability differently (Dunn & Andrews, 2015). Therefore, putting the person before the disability is essential, and this can be achieved by person-first language (PFL). The minority model portrays disabilities as neutral (Olkin & Pledger, 2003) and just as the social model, it is concerned about prejudice and discrimination. However, its response to these negative reactions is notably different. It claims that separating the disability from the individual might increase rather than decrease stigmatization, because doing so could inadvertently strengthen its negative connotations. For example, qualitative research has found that PFL is most often used in scholarly writing about the most stigmatized disabilities, which suggests that using PFL may actually accentuate the stigma (Gernsbacher, 2017). Moreover, PFL would not acknowledge that one’s disability can be central to one’s sense of self, and thus, a source of pride and celebration (Andrews et al., 2013). Unlike the social model, the minority model emphasizes the communities that disabled people are part of (Dunn & Andrews, 2015) and stresses the importance of these communities for creating a sense of belonging and a positive social identity (Brown, 1995; Gill, 1995). Accordingly, it advocates the use of identity-first language (IFL) to support the positions of disabled people. Importantly, both the social model and the minority model revolve around questions of identity. Yet, whereas the former stresses the importance of people’s *personal* identities (what makes them unique and different from other individuals) the latter stresses the importance of their *social* identities (what they share with ingroup others and distinguishes them from outgroup others) (see Turner et al., 1987).

### Preferences and Reasons

Most quantitative studies on differences in language preferences within the autism community, and the people around it, involve the English language. Research in Australia (Bury et al., 2020), the UK (Kenny et al., 2016), the US (Taboas et al., 2022), and the UK and the US combined (Kapp et al., 2013), has shown that most of their autistic respondents preferred IFL to PFL, and the same holds for a study among e-learners in a worldwide online course on autism education (Lei et al., 2021)^1^. Some of these studies have also included respondents around the autism community and found that this preference was less outspoken among friends and family and even reversed among professionals (Kenny et al., 2016; Lei et al., 2021; Taboas et al., 2022). Despite these differences, there was no complete unanimity within the community in the countries studied. Tabaos et al. (2022) found that 87% of their autistic participants indicated to use IFL than PFL when forced to choose between the two. However, Kenny et al., (2016) found that, when given the opportunity to select multiple terms, 61% endorsed the term “autistic”, and 28% endorsed the term “person with autism”. Moreover, 45% endorsed the more neutral term “on the autism spectrum”. “Person on the autism spectrum” was found to be relatively popular by Bury et al., (2020) as well. The latter asked their autistic respondents to rate and rank several terms. These ratings showed considerable variation (standard-deviations ≥ 1.94 on a seven-point scale), and the authors concluded that “autistic” (IFL) was highly polarizing as many participants ranked it as least preferred and very few as in between.

As far as we know, there has been only one quantitative study on autism language preferences in the Netherlands, the context of our own study. Buijsman et al., (2022) examined autistic adults and parents of children with autism in the Netherlands using the Dutch language. Based on the available research in English-speaking countries, they expected to find a stronger preference for IFL versus PFL in both respondent groups. They found the exact opposite as, respectively 68.3% (vs. 22.73%) and 82.5% (vs. 11.9%) of the autistic adults and parents of autistic individuals preferred PFL (vs. IFL). However, they also found that the younger adults were more likely to prefer IFL, which might have to do with a stronger active involvement in social media conversations, and thus with more exposure to the debate in Anglophone contexts.

Two of the aforementioned studies used open-ended questions and qualitative analyses to examine participants’ explanations for their language preferences. These explanations were generally consistent with the model underlying the language they preferred. In line with the social model, respondents in Bury et al.’s (2020) study who disliked IFL were more likely to consider it as judgmental and prejudicial, arguing that the autism diagnosis is the first thing it refers to. Likewise, some of the answers of the autistic participants in Kenny et al.’s (2016) research were consistent with the minority model, as they included the argument that autism cannot be separated from the person and that it implies a different way of seeing the world rather than a disorder. Although both studies provided insights in the reasoning behind the language preferences of autistic individuals (see also Lei et al., 2021), they did not quantitatively examine the relations between these preferences and the endorsement of the arguments that underly them. Doing so is important, for two reasons. First, not all persons who prefer a particular language use may agree with the reasoning behind it. For example, one may prefer IFL, not so much because one thinks that stressing one’s individuality is important, but because one experiences normative pressure to use it. Second, people may favor one type of language use, not so much because of its perceived advantages, but because of the perceived disadvantages of the other type. For example, IFL may be preferred if PFL is seen to increase stigmatization (see Botha et al., 2022). Such possibilities can only be evaluated by measuring participants’ perceptions of the consequences of both language types.

The present study seeks to complement the earlier research by examining the perceived consequences of IFL and PFL among individuals in the Dutch autism community. We focused on two types of consequences that are central to both the social and the minority model. That is to say, they the degree to which IFL and PFL contribute to the reduction of prejudice against people from the autism community and the degree to which they do justice to the identity of these people. In line with the earlier studies and the literature on disability models, we anticipated that a more positive perception of a particular language type (IFL or PFL) would be related to a stronger preference for it. In addition to this, we tried to make another unique contribution to the literature by examining two factors that are theoretically relevant for understanding individual differences in the perceived consequences and evaluations of IFL and PFL within the autism community: participants’ degree of identification with this community and their relative age of receiving a diagnosis.

### Identification and Uncertainty

The possibility that different language preferences within the autism community can be explained by individual differences in consequence perceptions, raises the subsequent question where these different perceptions come from. In the present study, we addressed this question by examining the role of group identification and age of diagnosis, and by using a social psychological perspective that combines the Social Identity Approach (Reicher et al., 2010) with Uncertainty-Identity Theory (Hogg, 2007). The social identity approach comprises both Social Identity Theory (Tajfel & Turner, 1981) and its successor Self-Categorization Theory (Turner et al., 1987), and it provides a highly promising yet underutilized perspective to study disability identities (for an excellent review, see Dirth & Branscombe 2018). Uncertainty-Identity Theory is partly based on this approach and can be easily integrated with it (Hogg, 2007, 2014).

According to Social Identity Theory (SIT), the groups we belong to are important for our sense of self, because they determine our social identity, or “that part of the individual’s self-concept which derives from their knowledge of their membership of a social group (or groups), together with the value and emotional significance of that membership” (Tajfel, 1981, p. 255). SIT further claims that individuals have need for a positive social identity, which is undermined when the groups they belong to are disadvantaged and have low social status. Stigmatization, as in the case of disability, can lead to a negative social identity, and thus threaten the self. According to the theory, there are both individual and collective strategies for dealing with this. Whereas the individual strategies involve attempts to dissociate the self from the group, either behaviorally or cognitively, the collective ones include efforts to improve the value and meaning or actual position of the group as a whole, and thus imply a move toward the group (Blanz et al., 1998; Tajfel & Turner, 1979). To the best of our knowledge, SIT’s ideas about social identity management have not been applied to people’s evaluations of disability language. However, because PFL downplays the value of disability group membership, it can be analyzed as an individual identity management strategy for people with disabilities. Conversely, IFL stresses the value and meaning of this group membership, and preferring it based on its perceived positive consequences can be seen as a collective strategy to deal with a stigmatized social identity (see Dirth & Branscombe 2018).

Whereas SIT focuses on the implications and meanings of social identities, Self-Categorization Theory (SCT) examines the conditions under which they are salient. It claims that people can categorize themselves and others at different levels of abstraction with distinct cognitive effects. They can see themselves as unique individuals (personal identity), but also as group members (social identity), in which case group characteristics become self-defining. Whether a particular group categorization is active depends on context, but also on perceiver readiness, the degree to which individuals are prone to use it (Turner et al., 1994). One factor that contributes to this perceiver readiness is people’s group identification. That is to say, individuals who strongly identify with a particular group are more likely to see themselves as members of it (self-categorization) and to see others as either ingroup members (belonging to their group) or outgroup members (not belonging to their group) (see Schubert & Otten 2002). Hardly any studies have examined people’s group identification as a predictor of their social identity management strategies. Yet, based on the social identity approach it can be hypothesized that people prefer collective strategies if they strongly identify with the group, but individual strategies when they consider the group as unimportant for their identity (see Mummendey et al., 1999). Hence, in the present research we investigated whether a stronger identification with the autism community would be related to a stronger preference for IFL and a weaker preference for PFL, via the perceived consequences of these language types.

Social identities are not only relevant as sources of self-evaluation but serve other important functions as well. According to Uncertainty-Identity Theory, social identities provide meaningful perspectives from which to understand the world, and group identities can reduce uncertainty as they provide “a sense of who we are that prescribes what we should think, feel, and do” (Hogg, 2007, p. 80). This seems to hold for people with autism in particular, as they tend to be more intolerant of uncertainty (Hwang et al., 2020). Qualitative research suggests that the timing of one’s autism diagnosis is important in this respect. Lilley et al., (2021) and Leedham et al., (2020) investigated how people on the autism spectrum felt after receiving their diagnosis later in their life. They found that this was mostly experienced positively: Their respondents started to finally accept themselves, because it gave them access to a new identity which provided them with a framework to learn about themselves and improve their well-being. Many of them were proud that what they initially thought (e.g., that they were “weird”) could now be explained by a type of neurodiversity which they could celebrate (Leedham et al., 2020; Lilley et al., 2021). Consistent with this earlier research and Uncertainty Identity Theory, we therefore examined whether people who had received their autism diagnosis later in their lives would be more likely to value their autism identity and thus report a stronger preference for IFL and a weaker preference for PFL, again via the perceived consequences of these language types.

### Overview of the Present Study

The present study used a social psychological approach to examine individual differences in preferences for IFL and PFL within the Dutch adult autism community. We investigated these preferences as identity management strategies, focused on community identification and relative age of diagnosis as predictors, and examined the perceived consequences of IFL and PFL as mediating variables.

**Fig. 1:**
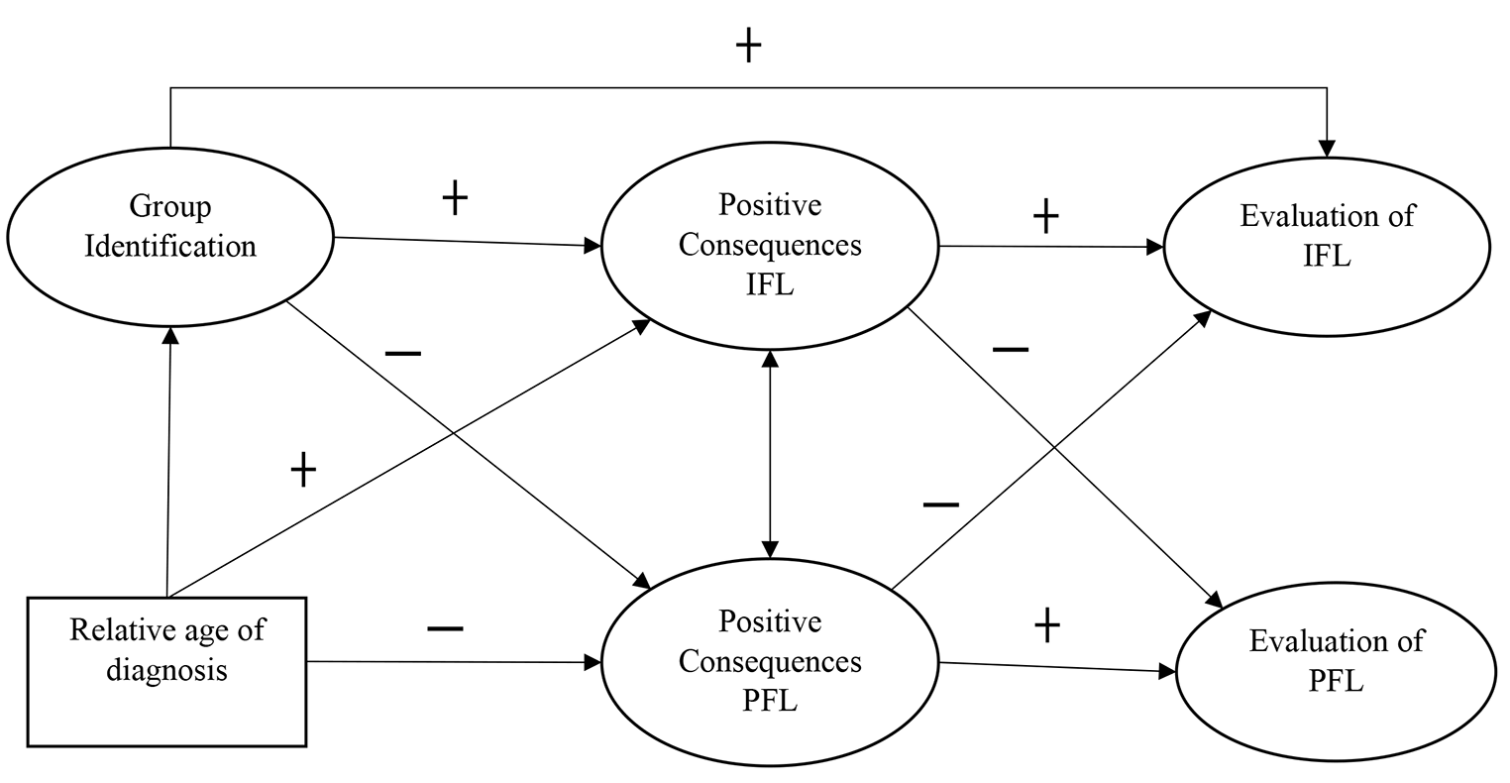
The hypothesized path model with the expected relations between the variables

Our hypotheses are summarized in Fig. 1. We tested them with Structural Equation Modeling (SEM) as will be explained below. Consistent with earlier research and the literature on disability models, we expected that a stronger perception of positive IFL consequences would predict a more positive evaluation of IFL (Hypothesis 1), and that a stronger perception of positive PFL consequences would predict a more positive evaluation of PFL (Hypothesis 2). However, we also explored whether the perceived consequences of IFL were related to a preference for PFL and vice versa. Next, we tested whether both a stronger identification with the autism community and a later age of diagnosis would predict a stronger perception of positive IFL consequences (Hypotheses 3a and 4a) and as a result, a stronger preference for IFL (Hypotheses 3b and 4b). Likewise, we anticipated that identification and later age of diagnosis would be associated with a weaker perception of positive PFL consequences (Hypotheses 5a and 6a), and therefore indirectly with a lower preference for PFL in return (Hypotheses 5b and 6b).

We also explored the possibility that these expected effects of participants’ relative age of diagnosis ran via their identification with the autism community. However, we had no a priori expectation about the relation between these two variables. It could be argued that the acquisition of a new meaningful identity leads to a stronger sense of belonging to others with whom that identity is shared, implying a positive relation between relative age of diagnosis and community identification. However, it may also take time to develop a sense of connection to a community that one only recently “became” part of.

Finally, we asked participants to explain their preferences in words – to examine whether their answers matched the results of our quantitative analyses – and to indicate their perceptions of the general level of prejudice against people with autism – to test our assumption that autism involves a stigmatized identity.

## Method

### Participants and Procedure

The data was collected in the Netherlands between May 5th 2022 and May 16th 2022 through an online survey via Qualtrics software. Ethical approval for this study was obtained from the Ethics Review Board of the authors’ institution (project no. 22-1225). Participation in this research was completely voluntarily, and all participants provided active informed consent. They were approached in two ways. First, a convenience sampling method was used by asking autistic adults within the first author’s network and contacted disability-advocating diversity networks to fill out and spread the survey. Next, snowball sampling was used by asking participants to share the link to the survey in their own networks. Initially, 313 people opened the survey but 215 of them completed it. The other individuals quit after reading the information letter (*N* = 15), providing informed consent (*N* = 34), completing the demographic questions (*N* = 7), or completing the identification items (*N* = 42) (see questions below). The final sample of 215 respondents had a mean age of 30.24 years (*SD* = 9.92; range 18–61), with 60 of them identifying as male and 138 as female, and 17 choosing the option “other/ I do not want to say” to indicate their gender. Two-hundred-and-fourteen participants completed primary school, 209 respondents finished high school, and 191 respondents followed another degree after high school. The different educational levels were all well represented among the respondents.

The survey consisted of 19 items and 2 open-ended questions, which took a couple of minutes to fill in. Participants were allowed to stop at all times but could not skip items. After finishing the survey, the respondents received a thank you note for finishing the questionnaire. Moreover, the e-mail address of the researcher was given so respondents could ask their questions and share their thoughts about the survey or their participation.

### Measures

**Demographic questions.** The survey started with demographic questions about age, gender, and highest level of completed education.

**Perceived prejudice.** After the demographic questions, participants completed the item “I feel like a lot of people have prejudice against people with an autism diagnosis”. This item was developed for the present study and measured on a 7-point scale ranging from 1 (*strongly disagree*) to 7 (*strongly agree*). The mean score on was 6.30 (*SD* = 0.92) and 96.7% of the respondents scored above the midpoint of the scale (4). This implied that most participants experienced an autism diagnosis to be highly stigmatizing.

**Relative age of diagnosis.** Next, there was a question about the age at which participants had received an autism diagnosis. Measuring the age of receiving a diagnosis was also our way of ensuring that respondents were diagnosed autistics. Moreover, in the information letter this was also mentioned as a requirement to participate. As age of diagnosis was strongly correlated to chronological age, *r* = 0.79, we calculated its proportion to the latter. The mean score on this measure was 0.79 (*SD* = 0.24, range 0.12 -1.00)^2^ which indicates that, on average, participants had spent almost 80% of their lives without an autism diagnosis.

**Community identification.** To measure the degree of participant’s identification with the autism community, we adapted four items that have been successfully used in research on ethnic group identification (e.g., Thijs et al., 2016): “I feel connected to the autism community”, “I am proud to be part of the autism community”, “Being part of the autism community is **not** an important part of who I am”(R), and “I feel involved in the autism community”. The items were measured on a 7-point scale ranging from 1 (*strongly disagree*) to 7 (*strongly agree*). Cronbach’s alpha was 0.77 for them.

**Perceived consequences of PFL and IFL.** To assess the perceived consequences of PFL or IFL we used two items for each of them: “I think that [person-first language/identity-first language] contributes to a decrease of prejudices about people in the autism community” and “I think that [person-first language/identity-first language] does justice to the identity of people in the autism community”. Prior to these items the difference between IFL and PFL was explained (see Appendix). The response scale ranged from 1 (*strongly disagree*) to 7 (*strongly agree*). Both items were developed for this study and reflected the central arguments in the debate about PFL versus IFL (see Dunn & Andrews, 2015). They were strongly correlated, *r* = 0.71, for both PFL and IFL.

The item means and standard deviations are shown in Table 1 (upper half). One-sample t-tests indicated that the mean score deviated significantly from the scale midpoint for one item: Respondents generally agreed with the notion that IFL does justice to the identity of people in the autism community, *t*(214) = 5.05, *p* < 0.01. Moreover, paired-sample t-tests, showed that for both PFL and IFL, respondents agreed more with the perceived positive consequences for identity as compared to prejudice reduction respectively, *t*(214) = 3.15, *p* < 0.01, and *t*(214) = 6.80, *p* < 0.01.

**Table 1:** Means and Standard-Deviations for Perceived Consequences and Evaluations

	Person-First Language	Identity-First Language
*Perceived Consequences*:		
Prejudice Reduction	3.89 (1.85)	3.98 (1.70)
Justice to Identity	4.19 (1.72)	4.59 (1.70)
*Evaluations*:		
Self	4.49 (1.56)	4.00 (1.85)
Ingroup Others	4.35 (1.51)	3.80 (1.74)

**Evaluations of IFL and PFL.** Respondents’ evaluations of IFL and PFL were assessed with two questions each. These items were developed for the present study and involved self-directed and other-directed language: “How would you generally feel when someone talks [to you**/**about **others in the autism community**] in identity-first language. So, someone says [‘you are autistic’/they are autistic].” and “How would you generally feel when someone talks [to you/about **others in the autism community**] in person-first language. So, someone says [‘you have autism’/‘they have autism’].” Strictly speaking is ‘having autism’ not PFL but should ‘that is a person with autism’ be used. However, ‘having autism’ improves the readability and implies a form of PFL, therefore this phrasing is used in the questionnaire. Respondents could answer on a response scale ranging from 1 (really undesirable) to 7 (really desirable) and the items were strongly correlated for IFL, *r* = 0.77, and PFL, *r* = 0.80). After the two questions on their language evaluations, participants were asked to provide elaborations of their answers.

Means and standard deviations for evaluation items are shown in the lower part of Table 1. For the two items involving PFL, the mean scores deviated significantly from the scale mid-point. Thus, participants positively appreciated PFL directed to themselves, *t*(214) = 4.64, *p* < 0.01, as well as to others, *t*(214) = 3.44, *p* < 0.01. Also, paired-sample t-tests, showed that participants preferred both PFL and IFL more for themselves than for others, respectively, *t*(214) = 2.13, *p* = 0.034, and *t*(214) = 2.52, *p* = 0.012.

### Data Analytic Strategy

We used Structural Equation Modeling (SEM) in Mplus Version 8.5 (Muthén & Muthén, 1998–2017) to examine the relations between our variables and test our hypotheses. We relied on four fit indexes: the comparative fit index (CFI), the Tucker Lewis index (TLI), the root mean square error of approximation (RMSEA), and the standardized root mean residual (SRMR). Model fit is considered good if CFI and TLI have values of 0.95 or higher, and RMSEA and SRMR are lower than 0.05. CFI and TLI values larger than 0.9 and RMSEA and SRMR values smaller than 0.1 are considered acceptable (Kline, 2011). As our SEM analyses involved latent factors for identification, perceived consequences (of IFL and PFL, respectively), and language preferences (for IFL and PFL, respectively), we first examined the factor structure behind the items for those measures.

## Results

### Factor Structure

We conducted Confirmatory Factor Analysis (CFA) and specified a five-factor model for (1) identification with the autism community, (2) perceived consequences of PFL, (3) perceived consequences of IFL, (4) evaluations of PFL, and (5) evaluations of IFL. This model showed an acceptable to good fit to the data, χ^2^(44) = 99.656, CFI = 0.958, TLI = 0.937, RMSEA = 0.077, SRMR = 0.053, and the correlations between the factors are shown in Table 2. Although the correlation between the perceived consequences of PFL and IFL was strong, *r* = -0.6 (Cohen, 1988), a four-factor model with a single factor for both consequences fitted the data significantly worse, χ^2^Δ(4) = 96.558, *p* < 0.01. Consistent with our first two hypotheses, there were positive correlations between participants’ perceptions of the positive consequences of each language type and their evaluations of it. However, there were also negative correlations between perceived consequences of a language type and the evaluation of the other type, and especially the relation between the perceived consequences of PFL and the evaluation of IFL was very strong, *r* = -0.8. Next, community identification was positively related to the preference for IFL, but unrelated to the preference for PFL. The two language preferences were negatively related, but the correlation was not strong.

**Table 2:** *Correlations between Latent Factors*

Variable	1	2	3	4
1. Group identification				
2. Positive consequences PFL	-0.33**			
3. Positive consequences IFL	0.33**	-0.60**		
4. Evaluation PFL	-0.12	0.60**	-0.39**	
5. Evaluation IFL	0.42**	-0.80**	0.61**	-0.44**

** *p* < 0.01

### Path Model

**Fig. 2:**
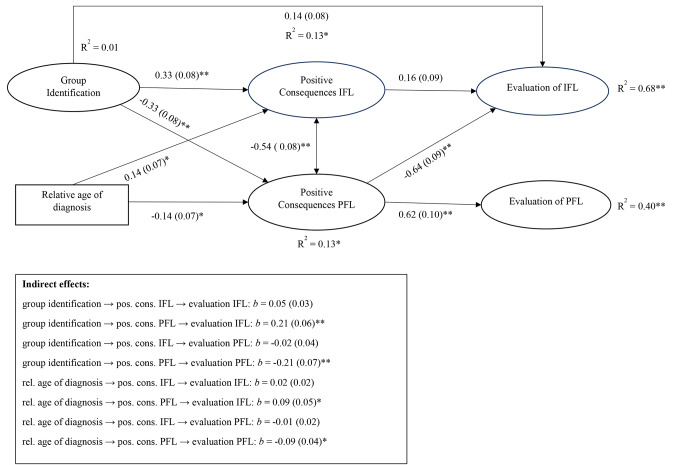
The tested path model with the relations between the variables along with the indirect effects

Next, we further tested our hypotheses using a path model with the abovementioned factors. That is to say, we regressed (a) evaluations, perceived consequences, and identification on relative age of diagnosis, as well as on the control variable gender (two dummy variables for ‘female’ and ‘other’), (b) evaluations and perceived consequences on identification, and (c) evaluations on perceived consequences. The fit of the model was acceptable to good, χ^2^(65) = 130.928, CFI = 0.951, TLI = 0.922, RMSEA = 0.069, SRMR = 0.048. Because this model included indirect effects, we used bootstrapping (1000 samples) to estimate standard-errors. The results of this model are displayed in Fig. 2. For readability purposes, direct effects with *p* > 0.1, and effects of gender (all *p* > 0.05) are not shown.

Group identification and relative age of diagnosis had no significant direct effects on the evaluations of PFL and IFL (and both variables were unrelated themselves as well). However, they did predict the perceived consequences of the two types of language: As expected (Hypothesis 3a), high identifiers reported more positive consequences of IFL and less positive consequences of PFL (Hypothesis 4a), and the same held for participants who received their diagnosis relatively late (Hypotheses 5a and 6a). The perceived consequences of IFL had no significant effects on the evaluations of IFL, which was not in line with Hypothesis 1, or PFL – despite its significant correlations with both variables in Table 2. However, individuals who perceived more positive consequences of PFL were more likely to desire PFL, which we expected (Hypothesis 2), and also less likely to desire IFL.

Figure 2 also shows that participants’ group identification and relative age of diagnosis predicted the evaluations of both types of language, but only indirectly via the perceived consequences of PFL. These effects were positive for the evaluation of IFL and negative for the evaluation of PFL. Thus, in line with Hypotheses 5b and 6b, both high identifiers and more recently diagnosed participants were less in favour of PFL, apparently because they disagreed with its positive consequences. However, this disagreement was also related to a more positive evaluation of IFL.

**Answers to the open-ended questions.** Finally, we investigated the answers to the open-ended questions to examine participants’ own explanations for the different language preferences. Initially, we tried to code whether participants referred to positive or negative consequences of IFL or PFL (or both) to explain their preference for each language type, but this proved to be hard as these distinctions could not always be made, and because sometimes respondents commented only on one language type.

Instead, we (the two authors) independently coded whether the available responses per person (110 participants responded) were (a) explicitly in line with the debate around IFL (and the minority model) versus PFL (and the social model), (b) explicitly in line with the debate but also indicating something else (“other”), or (c) indicated something else only. Because interrater agreement was moderate (Kappa = 0.53), we reinspected the cases we disagreed about (*n* = 32) and reached a unanimous decision.^3^ The final scores are shown in Table 3. They are organized by respondents’ quantitative evaluations of each language type, with “positive” meaning scores larger than 4 for both self and others, “negative” meaning scores smaller than 4 for both self and others, and “mixed” meaning all remaining scores.

**Table 3:** Explanations by Evaluation of Language Preferences

	Consistent with debate	Consistent with debate + other	Only other
*Identity-First Language*			
Positive	11 (34.4%)	9 (28.1%)	12 (37.5%)
Mixed	7 (14.9%)	9 (19.1%)	31 (66.0%)
Negative	8 (25.8%)	9 (29.0%)	14 (45.2%)
*Person-First Language*			
Positive	8 (18.2%)	12 (27.3%)	24 (54.5%)
Mixed	6 (13.6%)	12 (27.3%)	26 (59.1%)
Negative	12 (54.4%)	3 (13.6%)	7 (31.8%)
*Note.* Percentages involve rows.			

It appeared that participants who clearly preferred or disliked IFL used more arguments in line with the debate, and that participants with mixed evaluations of it were more likely to report other explanations but this association failed to reach significance, χ^2^(4) = 7.47, *p* = 0.113. However, there was a significant relation between participants’ evaluations of PFL and their explanations, χ^2^(4) = 14.85, *p* < 0.01. As shown in Table 3, respondents who rejected PFL were more likely to argue in line with the debate.

Finally, we discuss some explanations in detail. The quotes below were translated from Dutch to English. Different explanations were given for the rejection of PFL which were in line with the current debate. Respondents described autism as an inseparable part of who they are and mentioned that IFL endorses this better than PFL. They also stated that it is a big part of their life and how they experience the world.

“*My autism is a part of who I am. I was born with it, it is inseparable from who I am. Identity-first language endorses this for me*.”

“*Autism is who I am. I can be a person with red hair, a person with a nose-piercing or a person with a broken leg, but I am autistic. It is who I am, not just a part. It is how my brain functions, something that will not change*.”

For the preference for PFL other reasons were given, which were also in line with discussed debate. Some of the most reoccurring reasons were that respondents are more than their autism and that this is just as important.

“*Autism is just a small part of who I am. I have autism, this is a part of who I am, but does not say everything about me*.”

“*Identity-first feels, for me, very limited in how others view me, as if my autism is the only important thing about me*.”

Some explanations where not in line with the discussion and it is important to mention the nuances that were brought to light and cannot be seen in the path model. Respondents stated that context and connotations are important factors when looking at their preferences. They stated that this is the case when others use IFL for autistic individuals.

“*It is mainly the context in which it is being said whether it is desirable to use or not*.”

“*When I say it myself, I know what I mean. If another says it, I often get the idea that it has a negative connotation*.”

“*For me it depends on the intonation of how it is said. When it is said neutral, I think it is fine. When it is said in a judging manner it can be perceived as harmful*.”

It was also repeatedly mentioned that language preferences are personal matters and that it is important to look at individual differences.

“*The most important thing is that the person who it is about is comfortable with the language use*.”

There were also quite some respondents that stated that they do not care what terminology is used as long as it is used respectfully.

“*In my opinion the intention of speech (respect) is way more important than the spoken words*.”

## Discussion

The aim of this research was to obtain a better understanding of IFL and PFL preferences within the Dutch autism community by taking a social psychological perspective. Although we assessed this with one item only, almost all of our participants perceived prejudice against people diagnosed with autism. Hence, we could apply the Social Identity Approach (Reicher et al., 2010) and examine their preferences as collective (IFL) and individual (PFL) strategies for managing negative social identities.

Although we were interested in individual differences, we observed an overall preference for PFL over IFL in our sample. This finding is inconsistent with research in Anglophone contexts (Bury et al., 2020; Kapp et al., 2013; Kenny et al., 2016; Lei et al., 2021; Taboas et al., 2022) but in line with the only other Dutch study on this topic that we know of (Buijsman et al., 2022). Accordingly, there might be something about the context of the Netherlands that affects people’s ideas about autism language there. Specifically, one possibility that could be explored in future research is that IFL has negative connotations in Dutch, because swearing with diseases is relatively common in the country (Ruette, 2018). Still, it is important to note that there was considerable individual variation (as in the other studies) and, on average, no rejection of IFL. Moreover, the evaluations of IFL and PFL were negatively correlated, but not very strongly so, indicating the possibility of preferring both.

We anticipated that the variation in the evaluation of each language type could be explained by its perceived positive consequences. For PFL this was indeed the case, as participants who preferred it were more likely to think that PFL does justice to the identity of people in the autism community and decreases prejudice against them. However, despite a strong and positive correlation between the perceived consequences of IFL and its evaluation (*r* = 0.6), in our path model, the latter was ultimately explained by the lack of perceived positive consequences of PFL. Interestingly, the opposite was not found for PFL, as its evaluation was not predicted by a lack of perceived IFL consequences. We can only speculate about the causes of this asymmetry, but it seems to reflect the history of the debate on disability language and more specifically the fact that the appeal to use IFL is a reaction to the call to use PFL (Dunn & Andrews, 2015). Participants who preferred PFL endorsed the reasoning behind it (social model), but the choice for IFL was ultimately a negative one, based on their lack of satisfaction with the language as currently prescribed and expected by many organizations.

We could not directly observe this pattern in participants’ answers to the open-ended questions, as it was difficult to distinguish the satisfaction with one language type from their dissatisfaction with the other. However, we did find that participants who disliked PFL were more likely to use arguments from the debate around IFL versus PFL, which is consistent with our above interpretation that this debate revolves around the negative evaluation of the latter. Arguably, these arguments may have been primed by our questions about perceived consequences, but they were less often referred to by the other respondents. Additionally, many participants gave other comments, some of which provided considerable nuance to the debate. We will consider a few of those when discussing the implications of our study (see below).

To further examine the individual differences in participants’ language preferences, we took into account their community identification and relative age of diagnosis. As expected, the former was positively related to their IFL preference, but negatively to their PFL preference. This is in line with the Social Identity Approach (Reicher et al., 2010), which would predict that high identifiers would pursue collective identity management strategies, whereas low identifiers would pursue individual management strategies. Likewise, we found that respondents who had received their diagnosis later in their lives were more likely to prefer IFL and less likely to prefer PFL. This is consistent with Uncertainty-Identity Theory (Hogg, 2007) and existing research showing that discovering one’s autism identity later in life can be an enriching and positive experience, because it increases self-understanding and self-acceptance (Leedham et al., 2020; Lilley et al., 2021). The effects of both identification and relative age of diagnosis were fully mediated by the perceived consequences of IFL, indicating that the preferences of higher versus lower identifiers and later versus earlier diagnosed participants were based on deliberate considerations.

Importantly, the effects of relative age of diagnosis were not mediated by community identification as both factors were unrelated in our sample. This might come as a surprise, given our theorical reasoning about the psychological relevance of the autism identity for people who recently acquired it. It is important to note, however, that our identification measure captured the belonging aspect of the autism identity (e.g., “I feel connected to the autism community”) more so than its psychological centrality (e.g., “Having autism is an important part of who I am”) (for an overview of identification aspects, see Ashmore et al., 2004). Arguably, it takes time to develop a sense of belonging to a newly “discovered” community, even if one’s autism is central to one’s sense of self. Future research could examine different identification aspects, and we suspect that the presently obtained effects for relative age can be largely explained the centrality of the autism identity. Remarkably, relative age of diagnosis was also strongly related to actual age at the time of participation, which means that older respondents received their diagnosis later in life than younger respondents. We do not have a clear-cut explanation for this. Yet it is consistent with a worldwide increase in the prevalence of autism between 1990 and 2019, presumably due to growing diagnostic attention for it (Solmi et al., 2022).

Taken together our results further demonstrate the value of the Social Identity Approach for understanding disability identities (see Dirth & Branscombe 2018). Apparently, it is meaningful to conceive of the IFL and PFL preferences in the autism community as, respectively, collective and individual identity management strategies. And this allows for the examination of concrete hypotheses about the broader effects of autism language, and disability language more generally. More specifically, the Social Identity Approach suggests that different identity management strategies can have their own risks and gains. Collective strategies can increase awareness and group acceptance, and stimulate social change, but there is also the risk of emphasizing and essentializing group differences. By contrast, individual strategies may have individual benefits but may (unintentionally) legitimize existing inequalities and not improve the situation of the group as a whole (see Dirth & Branscombe 2018). Future research could examine these potential risks and benefits for IFL and PFL, and study the conditions under which they are more likely. The results of those studies could provide meaningful input for the debate on disability language. Still, as mentioned in our introduction, research cannot settle this debate, and ultimately it is up to those directly involved how to weigh the collective and individual risks and benefits of IFL and PFL.

### Limitations

The present research has a number of limitations which should be considered when interpreting its results. First, our findings are based on cross-sectional data, which means that no causal relations between variables could be determined. We cannot rule out the possibility, for example, that participants’ language preferences affected their sense community identification. Still, it is highly unlikely that participants’ preferences determined their age of diagnosis, and our analyses and interpretation of the direction of effects were consistent with our social psychological approach.

Second, as the targeted group was hard to reach, we relied on convenience and snowball sampling. Our group of participants is not fully representative for the Dutch autism community. There were more women than men in our sample, which is inconsistent with the diagnostic rates of ASD (Loomes et al., 2017), and it is not clear if the entire autism community (e.g., non-verbal individuals) was reached. The use of a convenience sample through disability advocacy groups might have influenced the results, since these individuals might be strongly involved in the autism community. Still, we found meaningful variation in our identification measure.

Third, our research took place in the Netherlands. The results might be generalizable to other western countries (even if there are differences in overall preferences), but probably less so for countries where there is less knowledge about autism or more stigma around it, for example Korea (Kim, 2012) or Lebanon (Gillespie-Lynch et al., 2019). In those contexts it might be more difficult to identify with the autism community, and IFL and PFL might have different connotations there.

Fourth, although language use was the very topic of this study, we had to resort to particular language to measure the perceptions, experiences, and preferences of our participants. We chose to use the term “autism community”. Some respondents experienced this term as leading, since it suggested that they are part of the group individuals with autism, which they might not want to be a part of. However, only 3 of the 215 respondents expressed this concern and there was meaningful variation in the community identification variable. Related to this, in asking respondents about their language preference we did not directly use the formulations “autistic person” and “person with autism”. Thus, we did not refer to IFL and PFL in the literal sense. However, our formulations clearly implied these language types (see, for example, the title of Bury et al., 2020), and people will typically use them in colloquial language.

Lastly, we used only one item to measure participants’ perceptions of prejudice against people diagnosed with autism. The scores on this item should be interpreted with care as we do not have any information about its psychometric qualities, and as it was placed before the questions on identification, IFL and PFL, it could have influenced the responses to those questions by priming them with the concept of prejudice. Still, our measure has face validity, and it is not uncommon to use single item measures for concepts such as prejudice and discrimination, also in relation to disabilities (see e.g., Itzick et al., 2018). Moreover, the finding that our respondents strongly agreed with the item suggests that they were generally aware of the prejudice against people from the autism community.

### Implications and (Additional) Suggestions for Future Research

Our findings have a number of implications for describing and addressing people from the autism community. First, the variation in language preferences within this community indicates that neither IFL nor PFL will be acceptable or desirable for all people who are part of it. It might be productive to honor this variation, by asking individuals with autism about their own preferences, and if that is not possible, for example in written texts, by showing awareness of different language connotations and by being explicit about one’s intentions (as we tried to do in this study). Indeed, several respondents explicitly acknowledged that “no size fits all” by mentioning that their own preferences could be different from those of others. Second, the lack of disagreement about PFL and IFL implies that it is important to look for alternatives that are acceptable from both the social and the minority model perspective. An example of this could be “someone on the autism spectrum” which was highly preferred in the research of Bury et al., (2020) and mentioned by one of our own respondents. It stresses the importance of autism for one’s identity but also acknowledges that everyone with autism is unique. Our finding that proponents of IFL were ultimately driven by their negative perceptions of PFL suggests that there could be more attractive alternatives to them.

In addition to this, there are two suggestions for future research beyond those already mentioned. To begin with, it would be interesting and relevant to examine different language preferences for and within other disability groups. It may matter, for instance, whether disabilities are acquired or congenital (Dunn & Andrews, 2015). Yet, although we do anticipate mean differences between the language preferences of different disability groups, we expect them to be meaningfully related to the importance of the group identity. Next, future research could delve more deeply into the role of situational characteristics. More specifically, how people from the autism community respond to IFL or PFL may depend on how, when, and by whom these language types are used. For example, some participants suggested that IFL may be regarded as less stigmatizing when used by ingroup members (autism diagnosis) as compared to outgroup members (no autism diagnosis), and nonverbal aspects such as intonation and body language may matter as well.

## Conclusion

Despite its limitations and the work still to be done, this research makes a unique contribution to the literature by being one of the few outside the Anglophone context and using a social psychological approach to explain language preferences within the autism community. We showed that there is considerable individual variation in these preferences that can be meaningfully predicted by differences in perceived consequences, community identification, and relative age of diagnosis. This means that IFL and PFL are both desirable and undesirable depending on the person and their reasons. Ultimately, both language types should be used with respect and the awareness that some people might prefer one to the other.

It is important to listen to the group you are talking to and about. Policy makers can use the results of this research to improve their policies regarding the use of IFL and PFL. They should realize that doing “nothing about us without us” as said by Charlton (1998), a disability rights activist, also holds for communication about people with autism.

### Electronic Supplementary Material

Below is the link to the electronic supplementary material.

Supplementary Material 1

## Footnotes

1 Strictly speaking this was a qualitative study. However, the authors provided frequencies of their codes.

2 For one respondent the age of diagnosis exceeded their own age. We assumed that the former was similar to the latter in that case. Analyses without this respondent yielded the same results as those reported in the text. Results are available on request.

3 We disagreed mostly about explanations focusing on hurt and insults only. We finally coded them as “other”.
