# Supplementary material for: Language Preferences in the Dutch Autism Community: A Social Psychological Approach
Source: J Autism Dev Disord. 2023 Feb 9;54(5):1727–39. doi: 10.1007/s10803-023-05903-0 (PMC11136701; doi:10.1007/s10803-023-05903-0)
Supplement: Supplementary file 1 — Supplementary Material 1 [file 10803_2023_5903_MOESM1_ESM.pdf]

Survey information and questions

Information text

Bachelor thesis – inclusive language for people with autism

Information about the research

I am XX, a third-year student Interdisciplinary Social Sciences at XX University. In this research project I will look at how the Dutch autism community views their diagnosis and whether this is related to a certain linguistic preference. I want to do this with the autism community, since a lot of the time there is a lot said about them instead of discussed with them. Your opinion on this is really valuable!

The survey

In the first part you will be asked some questions that give a broad context to who is filling in the survey. This way I can see whether the research is representative, and the conclusions are applicable to a broader group.

The second part will consist of four questions that will investigate how you view your autism diagnosis and your relation to the autism community. In the last part there will be a few questions about your linguistic preference. There are no wrong answers in any part of the survey, I just want to know your preferences and believes.

Content warning

This research is about linguistic preferences, and what it relates to. Existing research suggest that everyone prefers something different, that is why for a large part I will talk about the autism community. This is the group of people who have an autism diagnosis. In the part of the questionnaire different perceptions of talking about autism as a diagnosis will be discussed that some people might experience as stigmatizing or offensive. I would like to make clear that none

of these terminologies reflect my own perspective in the research and they are there for research purposes only.

Who can fill in the survey?

Any Dutch adult above 18 with an autism diagnosis.

How much time will it take to fill in the survey?

Filling in the survey will only take a couple of minutes.

Additional information

Filling in the survey is completely anonymous. I will not be able to trace your answers back to you. You can end the survey at any time, not completely filled in surveys will not be used in the final research. For any other questions or concerns please contact me, this can be before, during or after the survey. If you would like to see the results, please also send me a message and I will share them once my bachelor project is finished. This can be done through my e-mail address XX

In advance, thank you for your time!

XX

Informed consent

Thank you for your interest in this research. Before the start of the survey, I would like to provide you with some more information about the research.

I - Background of the research

I, XX a bachelor student from XX University, will do research about autism diagnosis and linguistic preferences. I hope to explain with this research that there is no right answer, and that there are reasons for certain preferences that are more important than generalizations of those preferences.

II – Procedure

By accepting the terms of this research your data can be used and on the next slides of the survey you will find the several parts mentioned before. So, at first the part with some general questions, then the part with 4 questions about how you view your autism diagnosis and then the last part about your linguistic preferences. The last part has a short explanation about different preferences, so you have an idea what is meant with the terminology. There are no wrong answers and after you fill in the entire survey you will find a thank you note. If you would like to see the results or if you have any questions, you can contact me at XX. The final research will be written in Englisch, but on request a short summary can be provided in Dutch as well.

III – Risk

There are no direct risks when participating in this research. This research is approved by the ethical committee of the University. However, as mentioned, some statements might be perceived as offensive or stigmatizing.

IV – Benefits

By participating in this research, you help bring some perspective in the current autism research from the perspective of the autism community. This is important because often research is done

about a group without letting the group participate and have a say in the matter. However, there are no direct benefits for yourself by participating.

V – Confidentiality

The survey is completely anonymous, and no answers can be traced back to an individual. And the results can only be found in protected environments of XX University that only I have access to.

VI – Voluntary participation

You are not mandated to participate in this research, and it is completely voluntary.

Consent

By participating in this research, I understand that;

- My answers are anonymous.
- My answers will be used for the research of XX, Bachelor student at the University of XX.
- For any questions or results I can contact XX at XX
- There are no risks connected to participating in this research.
- My participation is voluntary.

o I have read the consent form and agree to using my data for this research.

Demographic questions

- What is your age in years?
- What is your gender?

- At what age did you receive your autism diagnosis?
    - Please give the best possible estimate if you do not know the exact answer.
- What is your highest achieved educational attainment?
- I feel like a lot of people have prejudices about people with an autism diagnosis. (scale)

Group identification scale

The statements below are about how you see yourself with regards to the autism community. With the autism community we mean the group of people that have an autism diagnosis. Please tick the box that applies to you the most where 1 means strongly disagree and 7 means strongly agree

1. I feel truly connected to the autism community.
2. I am proud to be part of the autism community.
3. Being part of the autism community is **not** an important part of who I am. (R)
4. I feel involved in the autism community.

Preference for IFL or PFL

There are different ways to talk about an autism diagnosis, as the introduction of the research suggested. The two main ways are identity-first language and person-first language, within this research we will look at the difference in preference and to what extend this is related to how you perceive your autism diagnosis.

When we talk about person-first language we mention the individual behind the diagnosis first, so you are someone who has autism (or: *I have autism*).

When we talk about identity-first language we talk about the diagnosis first, so we say someone is autistic (or: *I am autistic*).

- I think that person-first language contributes to a decrease of prejudices about the people in the autism community.

- I think that person-first language does justice to the identity of people in the autism community.
- I think that identity-first language contributes to a decrease of prejudices about the people in the autism community.
- I think that identity-first language does justice to the identity of people in the autism community.

Please fill in the scale how desirable you think these situations are.

- How would you generally feel when someone talks to you in identity-first language. So, someone says ‘you are autistic’.
- How would you generally feel when someone talks about **others from the autism community** in identity-first language. So, someone says ‘they are autistic’

Could you elaborate on your answers?

- How would you generally feel when someone talks to you in person-first language. So, someone says ‘you have autism’.
- How would you generally feel when someone talks about **others from the autism community** in person-first language. So, someone says ‘they have autism’

Could you elaborate on your answers?
